# CITED2 Binding to EP300 Regulates Human Spermatogonial Stem Cell Proliferation and Survival Through HSPA6

**DOI:** 10.1155/sci/2362489

**Published:** 2025-04-24

**Authors:** Yongzhe Chen, Bang Liu, Sisi Tao, Lvjun Liu, Jianxin Gao, Ying Liang, Weilei Dong, Dai Zhou

**Affiliations:** ^1^Gynecology and Obstetrics and Reproductive Medical Center, The First Affiliated Hospital, Hengyang Medical School, University of South China, Hengyang, Hunan 421001, China; ^2^MOE Key Lab of Rare Pediatric Diseases, University of South China, Hengyang, Hunan 421001, China; ^3^Hunan Provincial Key Laboratory of Regional Hereditary Birth Defect Prevention and Control, Changsha Hospital for Maternal and Child Health Care Affiliated to Hunan Normal University, Changsha, Hunan 410000, China; ^4^Institute of Reproduction and Stem Cell Engineering, School of Basic Medicine Science, Central South University, Changsha, Hunan 410000, China; ^5^Research Department, Reproductive and Genetic Hospital of CITIC-Xiangya, Changsha, Hunan 410000, China

**Keywords:** CITED2, EP300, HSPA6, proliferation, spermatogonial stem cells, testis

## Abstract

Spermatogonial stem cells (SSCs) are essential for the initiation and continuation of spermatogenesis, a process fundamental to male fertility. Despite extensive studies on mouse SSCs, the mechanisms governing self-renewal and differentiation in human SSCs remain to be elucidated. This study investigated the regulatory mechanisms of SSCs by analyzing single-cell sequencing data from the GEO dataset of human testis. Analysis revealed dominant expression of CITED2 in human SSCs. Reduction of CITED2 levels in hSSC lines significantly inhibited proliferation and increased apoptosis. Protein interaction prediction and immunoprecipitation identified interactions between CITED2 and EP300 in SSC lines. RNA sequencing results indicated that CITED2 knockdown significantly affected the MAPK pathway and the HSPA6 gene. Overexpression of HSPA6 mitigated the proliferative and apoptotic changes provoked by CITED2 downregulation. These findings provide novel insights into the regulatory and functional mechanisms of CITED2-mediated hSSC development.

## 1. Introduction

Infertility represents a prevalent worldwide health issue impacting around 15% of couples attempting to achieve pregnancy, with male factors constituting around 50% of these instances [1]. Nonobstructive azoospermia (NOA) is a severe and challenging medical condition of male infertility with a highly intricate etiology, greatly impacting the available therapeutic modalities [2]. Although a few NOA patients may be able to successfully retrieve sperm using microsurgical testicular sperm extraction (mTESE) and later employ assisted reproductive technology to conceive, the majority of NOA patients are unable to have biological offspring [3, 4]. Meeting the reproductive requirements of patients with NOA is a considerable clinical obstacle. Stem cells can undergo self-renewal, thereby restoring the functionality of tissues and organs. Transplanting spermatogonial stem cells (SSCs) in mice has been demonstrated to restore the reproductive lineage [5]. Additionally, the successful birth of offspring has been achieved through the technique of obtaining spermatozoa via *in vitro* cell culture in mice [6]. However, the clinical application of this experiment remains inaccessible to humans owing to the limited *in vitro* hSSC proliferation capability.

SSCs, located near the testicular seminiferous tubules' basal membrane, are regulated by both the microenvironment and intrinsic factors [7, 8]. Research has shown that glial cell line-derived neurotrophic factor (GDNF) participates in maintaining and enhancing SSC self-renewal [9]. Additionally, it has been discovered that a cell culture system, when supplemented with GDNF, fibroblast growth factor 2, and epidermal growth factor, may effectively support the long-term proliferation of mice SSCs *in vitro* [10]. While WNT signals are typically associated with the proliferation of undifferentiated spermatogonia (Undiff-SPG) [11], they have been reported to be involved in SSC differentiation into progenitor cells [12]. Several intrinsic factors, such as promyelocytic leukemia zinc finger (PLZF) [13], forkhead Nanos homolog 2, box protein O1 (FOXO1) [14], and inhibitor of DNA binding 4 (ID4) [15], contribute to governing SSC self-renewal and proliferation. RA may play a role in promoting SSC differentiation [16]. Furthermore, several molecules, including NEDD4 [17], SALL4 [18], STRA8 [19], and DOT1L [20], which play a role in promoting SSC differentiation.

Attempts have been undertaken to cultivate hSSCs *in vitro* based on findings from studies conducted on mice. However, the low proliferative capacity of human primary SPG has hindered their expansion *in vitro* for an extended period [21]. Given the differences in the classification of the germinal epithelial cycle and the molecular characteristics of the SSC between humans and mice, the developmental regulation of the hSSC may differ significantly from that of mice [22, 23]. Several molecules, such as OIP5 [24], MiR-1908-3 p [25], and MKK7 [26], have been shown to regulate hSSC proliferation. Previously, we reported that a number of molecules regulate human SSC development, including PTN [27], ASB9 [28] and MAGEB2 [29]. Nevertheless, these studies have not yet provided a comprehensive understanding of the mechanisms behind hSSC proliferation and self-renewal.

In this study, we conducted an integrated analysis of human testis single-cell sequencing data from GSE109037/120508 to explore the regulatory mechanism behind hSSCs [30, 31]. We detected a number of differentially expressed genes (DEGs) in hSSC, including ASB9, SPOCD1, and CITED2. Immunohistochemical (IHC) analysis results confirmed the localization of CITED2 in testicular tissue. Subsequent CITED2 knockdown experiments in hSSC lines demonstrated significant inhibition of proliferation and self-renewal. The results of immunoprecipitation (IP) revealed an interaction between CITED2 and EP300. Additionally, RNA sequencing analysis revealed that downregulation of CITED2 significantly impacted HSPA6 expression and inhibited the MAPK pathway. Notably, overexpression of HSPA6 rescued the proliferation inhibition of SSC lines caused by CITED2 downregulation. These findings enhance our comprehension of the regulation of early SSC formation and could guide the identification of therapeutic targets for male infertility.

## 2. Materials and Methods

### 2.1. Human Testis Tissue Collection

The Ethic Committee of the First Affiliated Hospital of University of South China authorized the current study (2023LL0828001), with all participants signing informed consent. The human testicular tissues were gathered from 20 patients (5 OA and 15 NOA) aged 25–46 years who received m-TESE, acquiring about 20 mg testicular tissues. After three washing processes with PBS containing 1% penicillin and streptomycin, the samples were fixed with 4% paraformaldehyde (PFA) or kept in liquid nitrogen.

### 2.2. Single-Cell RNA-Sequencing Data Analysis

The Seurat program (http://satijalab.org/seurat/, R package, version 4.3) was employed for six normal adult testis scRNA-seq dataset analyses. Three datasets were obtained from GSE109037 and three from GSE120508. Initially, we loaded expression matrix data onto R employing either the Read.table or Read.csv function, thereby developing Seurat objects from every assay. Thereafter, each assay filtration was followed by normalization per default settings. We only retained cells that exhibited the expression of >500 genes and had <20% of their readings mapped to the mitochondrial genome. After finding each object's variable features, all data were subjected to merging with the IntegrateData function. Following the removal of mitochondrial and ribosomal genes, UMAP and clustering analyses were conducted on the integrated dataset through the top 2500 highly variable genes and PCs 1–13. Furthermore, a more thorough analysis of the SSCs revealed the presence of two distinct subclusters. The UMAP plot was used to select SSCs, which were then analyzed for pseudotime using the “Monocle” in R (https://cole-trapnell-lab.github.io/monocle3/, version 3.0). The analysis started with the subcluster at State 0, generating the heatmaps in a sequential sequence based on pseudotime. Line plots were also created in the same pseudotime order, fitting curves via the “auto” method of the “ggplot2” package in R (https://ggplot2.tidyverse.org/).

### 2.3. hSSC Line Culture

The hSSC line was established by inducing the overexpression of the SV40 large T antigen in primary GPR125-positive Undiff-SPG [32]. This immortalized hSSC retains numerous characteristics of primary cells and is positive for multiple SSC markers: GFRA1, PLZF, UCHL1, and THY1. The cell line culture was performed in DMEM/F12 (Gibco, Grand Island, NY) that contained 10% FBS (Gibco) along with 100 units/mL streptomycin/penicillin (Invitrogen, CA, USA) in a 5% CO_2_ at 34 °C. Cell passage was conducted every 3 days utilizing 0.05% trypsin and 0.53 mM EDTA (Invitrogen) to detach and split the cells.

### 2.4. RNA Extraction and qPCR

Total RNA was extracted from the isolated cells using RNAiso Plus reagent (Takara, Tokyo, Japan), following the manufacturer's protocol. The quality and concentration of the extracted RNA were assessed using a Nanodrop spectrophotometer from Thermo Fisher Scientific. cDNA synthesis was performed using commercial kits from Roche (Basel, Switzerland), according to the supplier's instructions. Quantitative PCR (qPCR) was conducted on the ABI Prism 7700 system (Applied Biosystems) following the manufacturer's guidelines. Relative mRNA levels were determined using the 2-ΔΔCt method, with *β*-actin as the endogenous control. Each sample was analyzed in triplicate, and the average results were calculated. All primers were sourced from PrimerBank (https://pga.mgh.harvard.edu/primerbank/), and their sequences are provided in Supporting Information Table S1.

### 2.5. Gene Silencing

Small interfering RNAs (siRNAs; Ribobio, Guangzhou, China) were deployed for silencing specific genes in hSSCs. Per the protocol, the hSSC transfection with siRNA (100 nmol/L) was performed employing Lipofectamine 3000 (Life Technologies, Carlsbad, CA, USA). After 48 h, the cells were harvested for mRNA and protein analysis to allow the specific knockdown of target genes in hSSCs, thereby studying the effects of gene silencing on cell function and phenotype.

### 2.6. Immunohistochemical and Immunofluorescence (IF)

For immunohistochemical (IHC), testis sections were exposed to deparaffinizing with xylene and rehydration using graded ethanol. Antigens were unmasked through heat-induced antigen retrieval conducted in 0.01 M sodium citrate buffer at 98 °C for 18 min. Sections were treated with 3% H_2_O_2_ (Zsbio, Beijing, China) to block endogenous peroxidase activity and were treated with 0.25% Triton X-100 (Sigma) for 15 min, aiming at increasing membrane permeability. This was followed by locking with 5% BSA for 1 h at room temperature (RT) for the prevention of non-specific binding of antibodies. Thereafter, sections were incubated with primary antibodies (Supporting Information Table S2) at 4 °C for a whole night, followed by rinsing with PBS and incubation with HRP-labeled secondary antibody for 1 h at RT. Herein, we detected chromogen with the 3,3'-diaminobenzidine chromogen kit (Dako, Glostrup, Denmark), thereby staining the sections with hematoxylin.

For IF, the procedure included incubation with Alexa Fluor conjugated secondary antibody for 1 h at RT. This was followed by staining cell nuclei with DAPI and acquiring images via a Zeiss microscope. This detailed protocol outlines the steps taken to prepare and stain testis sections for IHC analysis, including specific treatments to enhance signal detection and preserve tissue integrity.

### 2.7. Western Blot and IP

Testis samples and hSSCs were homogenized and subjected to lysis on ice for 30 min employing Radioimmunoprecipitation assay (RIPA) lysis buffer (Thermo Scientific). The lysates were then centrifuged at 12,000*g* to gather clear supernatants, determining protein concentration in the lysates via a BCA kit (Thermo Scientific). Protein Co-immunoprecipitation (Co-IP) assays were conducted through the Pierce Classic Magnetic IP/Co-IP Kit (Thermo Scientific). In brief, primary antibodies or control rabbit IgG were introduced to the cell lysate and incubated for a whole night at 4 °C. Subsequently, we introduced protein G magnetic beads to the supernatants and allowed them to incubate at 4 °C for 2 hr. After three rinsing cycles with washing buffer, the beads were subjected to magnetic pellet, resuspension, and boiling at 95 °C for 5 min. Totally, 30 *μ*g of total protein extracts from each sample were exposed to SDS-PAGE (Bio-Rad), performing western blotting per the previously published protocol. Table S2 (Supporting Information) provides a comprehensive overview of the antibodies utilized in the study, along with their corresponding dilution ratios. Using chemiluminescence (Bio-Rad), the immunoreactive protein band intensities were visualized.

### 2.8. CCK-8 Assay

Post-siRNA transfection, the hSSC proliferation capability was assessed by the CCK-8 assay Kit (Dojindo, Kumamoto, Japan) per the protocol. Briefly, the cell growth medium was changed with a 10% CCK-8 reagent solution and incubated for 3 h. To measure the optical density (OD) values, a microplate reader (Thermo Scientific) was used at 450 nm.

### 2.9. EdU Incorporation Assay

Herein, 5,000 hSSCs/well were cultivated into a 96-well plate that contained DMEM/F12 medium supplemented with 50 *μ*M EdU (RiboBio) and allowed to incubate for 12 h. Subsequently, the cells were washed with DMEM and fixed with 4% PFA. For cell neutralization, 2 mg/mL glycine was used, followed by 10 min permeabilization with 0.5% Triton X-100 at RT. EdU immunostaining was carried out employing Apollo staining reaction buffer while utilizing Hoechst 33342 to stain the cell nuclei. Fluorescence microscopy (Zeiss) was employed to capture images, and a minimum of 500 cells were analyzed to determine EdU-positive cell proportion.

### 2.10. Flow Cytometry With Annexin V-APC/PI Staining

Aiming at evaluating downregulated CITED2 impact on hSSC line apoptosis, cells were subjected to digestion and ice-cold PBS wash twice. Per the protocols, 10^6^ cells were re-suspended in Annexin V Binding Buffer (BD Biosciences, NJ, USA). This was followed by cell incubation with 5 *µ*L of Annexin V-APC and 10 *µ*L of PI solution for 15 min at RT in darkness and analysis through a C6 flow cytometry (BD Biosciences).

### 2.11. RNA Sequencing

The Trizol reagent kit (Invitrogen) as well as the Agilent 2100 Bioanalyzer (Agilent Technologies, CA, USA) were deployed to isolate total RNA and measure RNA quality that was validated via RNase-free agarose gel electrophoresis. For the eukaryotic mRNA enrichment, the Oligo (dT) beads were utilized, while for prokaryotic mRNA enrichment, the Ribo-ZeroTM Magnetic Kit (Epicentre, WI, USA) was used to remove rRNA. The enriched mRNA was fragmented into short fragments through the fragmentation buffer and reversely transcripted into cDNA via Random Hexamers. Aiming at synthesizing the second DNA strand, DNA polymerase I, dNTP, buffer, and RNase H were utilized. Subsequently, we employed the QiaQuick PCR extraction kit (Qiagen, Venlo, The Netherlands) for the cDNA fragment purification, followed by end repair, introducing poly (A), and ligating with Illumina sequencing adapters. After that, agarose gel electrophoresis was carried out to select the PCR-enriched ligation products and sequenced on the Illumina HiSeq2500 system (Gene Denovo Biotechnology Co., Guangzhou, China). The sequencing-machine-acquired reads were filtered using fastp (version 0.18.0). The rRNA-mapped reads were eliminated utilizing the Bowtie2 short reads alignment tool (version 2.2.8). The remainder of the clean reads were subsequently deployed for assembly and to determine gene abundance. The THISAT algorithm was used with the option “rna-strandness RF,” and other default settings to align the paired-end clean reads to the reference genome. The StringTie v1.3.1 was utilized to construct the assembled reads using a reference-dependant approach. Assessing RNA differential expression was performed using the DESeq2 software, with an absolute fold change ≥2 and an FDR < 0.05 indicating DEGs.

### 2.12. Statistical Analysis

The dplyr R package (version 3.4.0) was employed for descriptive and statistical analyses. The experiment was conducted three times, representing the values as mean ± standard deviation (SD). The *t*-test was utilized to determine the statistical disparities between two groups, where *p* < 0.05 was deemed statistically significant.

## 3. Results

### 3.1. Single-Cell Sequencing Reveals the hSSC Transcriptome

**Figure 1:**
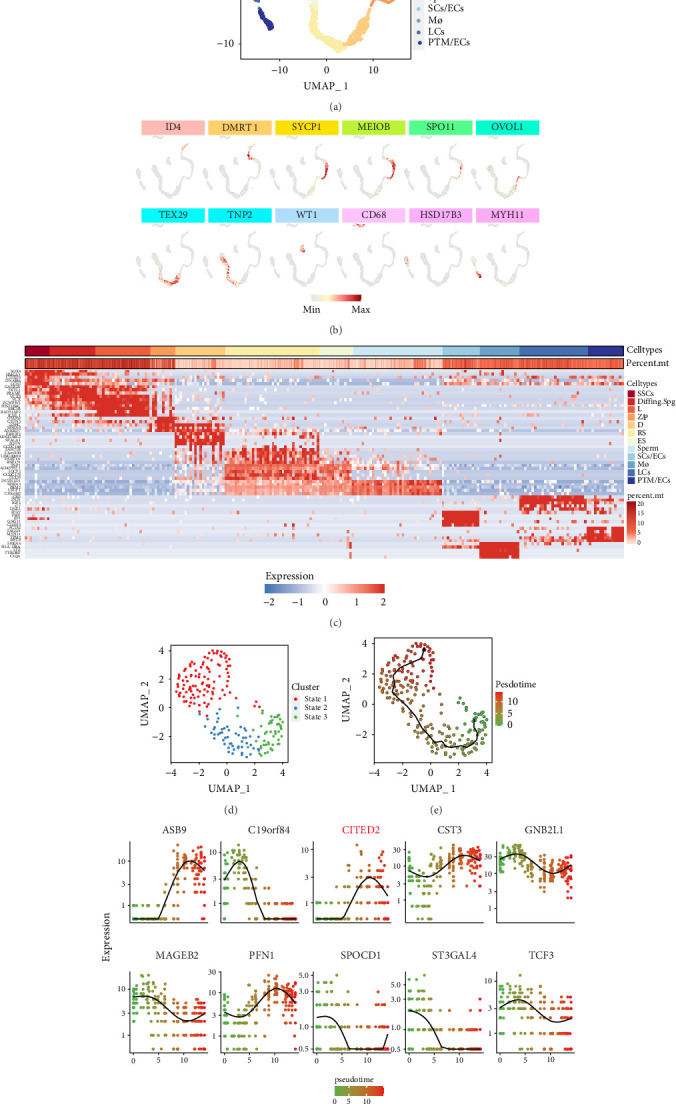
Single-cell sequencing data elucidating the transcriptomic landscape and developmental pathways of SSCs within the human testis. (A) UMAP clustering of human testicular cells. A total of 4937 cells were categorized into 12 clusters and colored as noted in the figure to the right, with each dot representing an individual cell. (B) Dot plot showing the projection of selected testicular markers on the UMAP plot. The scaled gene expression levels are colored according to the Z score at down. (C) Heatmap showing the level of the Top 5 differential genes in each cluster. The scaled gene expression levels are colored according to the Z score at down. (D) Reclustering of the SSC. All SSCs were divided into three different stages, including stages 2 and 3. Each dot represents a cell, colored according to the legend on the right. Arrows represent the direction of SSC development. (E) Constructing developmental trajectories of SSCs using Monocle3. The pseudotime of each cell was colored according to the legend at right. (F) Dot plots demonstrating changes in expression levels of Top 10 DEGs in SSC along with developmental trajectories. The pseudotime of each cell was colored according to the legend at down.

To elucidate the developmental trajectory of hSSCs, a meticulous analysis of several human testicular single-cell sequencing GEO datasets was conducted. After applying stringent quality control measures, a total of 4937 cells were retained, revealing the presence of 22,075 genes. Using Uniform Manifold Approximation and Projection (UMAP) clustering, these cells were classified into 12 distinct clusters. Consistent with prior findings, these clusters encompassed SSCs, differentiating spermatogonia (Diffing.spg), zygotene/pachytene spermatocytes (Z/P), diplotene spermatocytes (D), round (RS) and elongating spermatids (ES), spermatozoa (Sperm), Sertoli cells/endothelial cells (SCs/ECs), macrophages (Mø), Leydig cells (LCs), and peritubular myoid cells/ECs (PTMs/ECs). This classification was achieved by discerning the expression patterns of various testicular cell marker molecules (Figure 1A). The corresponding markers for these testicular cells, represented through dot plots (Figure 1B), include ID4, DMRT1, and SYCP1, among others. Furthermore, an in-depth analysis of the DEGs within each cluster was conducted, depicting the top 5 DEGs in a heatmap (Figure 1C). To delve deeper into the developmental trajectories of SSCs, data pertaining to the SSC cluster were exposed to further stratification, yielding three distinct stages (Figure 1D). Concurrently, the developmental pathways of SSCs were reconstructed using Monocle3 (Figure 1E). Subsequently, the top 10 DEGs associated with SSCs were superimposed onto these developmental trajectories to monitor their expression dynamics throughout the developmental continuum (Figure 1F). Notably, pronounced fluctuations in the expression levels of numerous genes during development were revealed. Intriguingly, the CITED2 gene exhibited a progressive upregulation coinciding with the differentiation process of SSCs, suggesting its potential role in modulating the fate determination of SSCs.

### 3.2. Expression Pattern of CITED2 in Normal Spermatogenesis Testis

**Figure 2:**
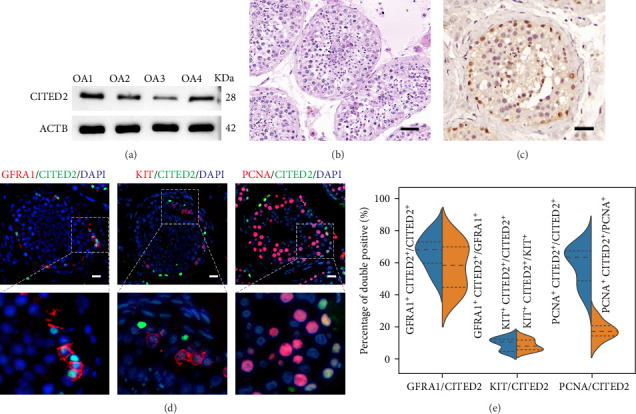
Distribution of CITED2 protein in the human testis. (A) Western blot detection of CITED2 protein expression in OA testis. (B) Representative images of HE staining from OA testes demonstrating normal spermatogenesis. Scale bar, 50 *μ*m. (C) The distribution of CITED2 in the testis was detected by immunohistochemistry. Scale bar, 50 *μ*m. (D) Immunofluorescence detection of CITED2 expression in SSC and Diff-SPG. Green represents CITED2 signaling, and red is SSC (GFRA1^+^), Diff-SPG (KIT^+^), and proliferating cell (PNCA^+^) markers, respectively. Cell nuclei were counterstained with DAPI. Scale bar, 20 *μ*m. (E) The violin plot demonstrates the positivity of CITED2 in SSC, Diff-SPG, and proliferating cells in D.

To validate CITED2 expression in bioinformatics analysis, four testes from patients having OA (normal spermatogenesis) who received mTESE were utilized. The presence of CITED2 in the testis was verified through a Western blot assay (Figure 2A). In testes with normal spermatogenesis (Figure 2B), CITED2 exhibited a nuclear localization in cells adjacent to the seminiferous tubules' basement membranes, imposing its predominant presence in SPG (Figure 2C). Two-color IF was employed to verify CITED2 distribution in SSCs and differentiated SPG (Diff-SPG). The results showed that CITED2 was mainly localized in GFRA1-positive SSCs, while a small amount of co-localization was also observed in Diff-SPG, consistent with the bioinformatics results (Figure 2D,E). Additionally, co-staining with PCNA showcased that CITED2 was mainly expressed in proliferating cells (Figure 2D,E). Collectively, CITED2 is predominantly expressed in proliferating SSCs.

### 3.3. Knocking Down CITED2 Inhibits Cell Proliferation and Induces Apoptosis

**Figure 3:**
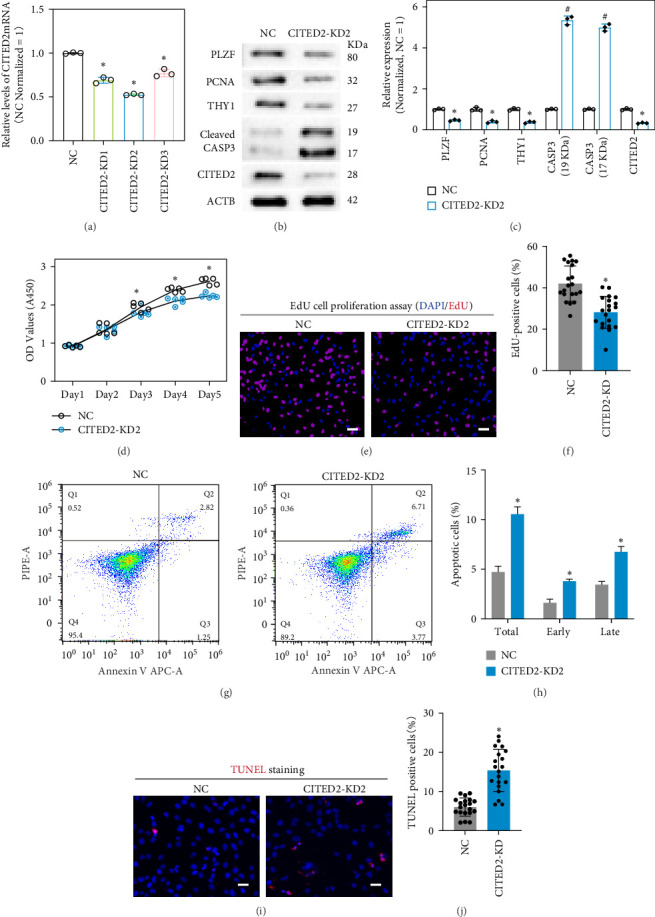
Influence of CITED2 knockdown on SSC line proliferation and apoptosis. (A) Bar graph demonstrating mRNA levels after CITED2 knockdown. (B) Western blot detection of SSC proliferation and apoptosis-related protein levels after CITED2-KD2 knockdown. SSC proliferation and self-renewal proteins, including PLZF, THY1, and PCNA, were significantly down-regulated upon CITED2 knockdown. Apoptosis-related protein CASP3 was significantly upregulated after CITED2 knockdown. (C) Bar plot demonstrating the relative expression levels of proteins relative to the NC group in B. (D) CCK8 detects cell proliferation 1–5 days after CITED2 knockdown. (E) EdU assay to detect DNA synthesis in cells after CITED2 knockdown. The red color represents EdU signaling, and nuclei were counterstained with DAPI. Scale bar, 20 *μ*m. (F) The bar plot demonstrates the EdU positivity of the cells in the E. (G) Apoptosis was detected by flow cytometry. (H) The bar graph demonstrates apoptosis in G. (I) TUNEL staining detects cells with broken DNA. Red color indicates TUNEL-positive cells with DNA breaks. Scale bar, 20 *μ*m. (J) Bar graph showing the proportion of apoptotic cells in I.*⁣*^*∗*^ represents *p* < 0.05, significantly downregulated compared to NC. # represents *p* < 0.05, significantly upregulated compared to NC.

CITED2 has been identified as a co-transcription factor implicated in stem and tumor cell proliferation and apoptosis [33]. To elucidate its role in hSSC development, a primary SSC-derived cell line was employed, and CITED2 expression was silenced using small interfering RNAs. qPCR and Western blot analyses demonstrated effective knockdown of CITED2 (Figure 3A,B). The Western blot results further indicated that proteins crucial for SSC proliferation and self-renewal, such as PLZF, THY1, and PCNA, were significantly diminished following CITED2 knockdown, whereas CASP3, a protein linked to apoptosis, was notably elevated (Figure 3B,C). The CCK8 assay revealed that CITED2 knockdown markedly suppressed cell proliferation (Figure 3D). EdU staining confirmed that DNA synthesis was hindered in cells post-CITED2 knockdown (Figure 3E,F). Moreover, flow cytometry-based apoptosis detection showed that both early and late apoptosis experienced a significant elevation upon CITED2 downregulation (Figure 3G,H). The results of TUNEL assay also revealed that CITED2 knockdown resulted in a significant increase in the percentage of cellular DNA breaks (TUNEL positive), suggesting that CITED2 downregulation promotes apoptosis (Figure 3I,J). Altogether, these results uncover the critical role of CITED2 in SSC proliferation and apoptosis.

### 3.4. CITED2 Functions in Combination With EP300

**Figure 4:**
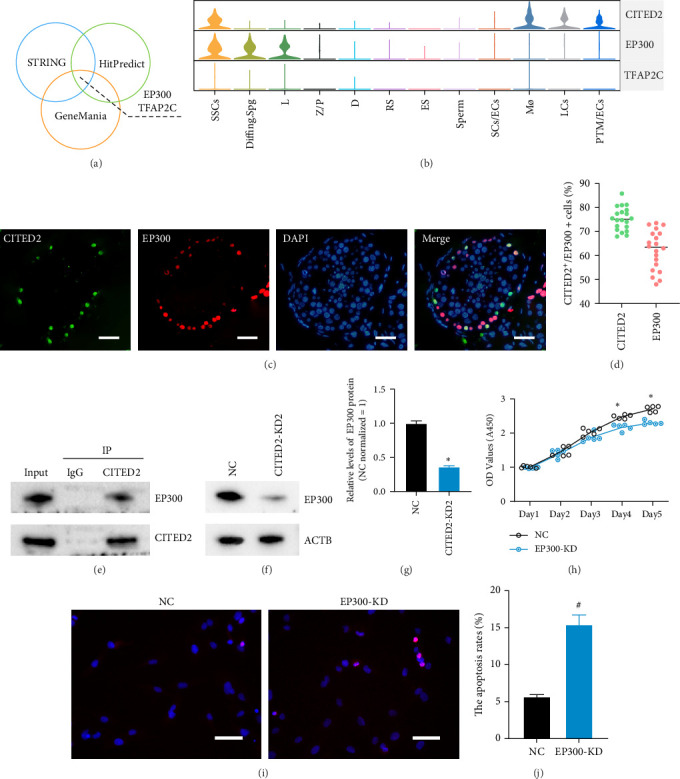
Prediction and validation of CITED2-binding proteins. (A) Prediction of CITED2-binding proteins. Three databases, including STRING, HitPredict, and GeneMania, were used to predict CITED2-binding proteins. The Wayne diagram shows the intersection of the three predicted data, EP300 and TFAP2C. (B) Violin plot demonstrating CITED2, EP300, and TFAP2C expression in the testicular single-cell transcriptome. (C) Immunofluorescence detection of CITED2 and EP300 localization in the testis. Scale bar, 50 *μ*m. (D) Dot plot demonstrating the rate of double positivity for CITED2 and EP300. (E) The co-IP assay detects the interaction of CITED2 protein with EP300 protein. (F) Western blot detection of EP300 expression level after CITED2 knockdown. (G) Bar graph demonstrating the expression level of EP300 after CITED2 knockdown. (H) CCK8 assay detects cell proliferation after EP300 knockdown. (I) TUNEL staining to detect apoptosis. The red color represents positive TUNEL staining with nuclei counterstained by DAPI. Scale bar, 50 *μ*m. (J) Bar graph showing the proportion of TUNEL-positive cells. *⁣*^*∗*^ represents *p* < 0.05, significantly downregulated compared to NC. # represents *p* < 0.05, significantly upregulated compared to NC.

To investigate its interacting proteins, we utilized three databases—STRING, HitPredict, and GeneMania—to predict its binding proteins. The results from these databases were intersected, revealing that CITED2 is most likely to bind to EP300 and TFAP2C (Figure 4A). In the single-cell transcriptome, EP300 is predominantly present in SSCs, Diffing.spg, and L, while TFAP2C expression is limited to testicular cells (Figure 4B). This suggests that CITED2 may bind to EP300 but not TFAP2C. Two-color IF in normal testes revealed that ~75% of CITED2-positive cells expressed EP300 (Figure 4C,D). Protein Co-IP assays were subsequently conducted to assess whether CITED2 indeed binds to EP300 and TFAP2C. The results demonstrated a specific interaction between CITED2 and EP300 within the human SSCs (Figure 4E), whereas no binding of CITED2 to TFAP2C was observed in these cells (data not shown). Additionally, the knockdown of CITED2 led to the down-regulation of EP300 expression (Figure 4F,G). Notably, following the knockdown of EP300, cell proliferation was significantly reduced (Figure 4H), while apoptosis levels were significantly increased (Figure 4I,J). This posits that EP300 interacts with CITED2 and jointly regulates SSC proliferation and apoptosis.

### 3.5. Screening of CITED2 Downstream Factors Using RNA Sequencing

**Figure 5:**
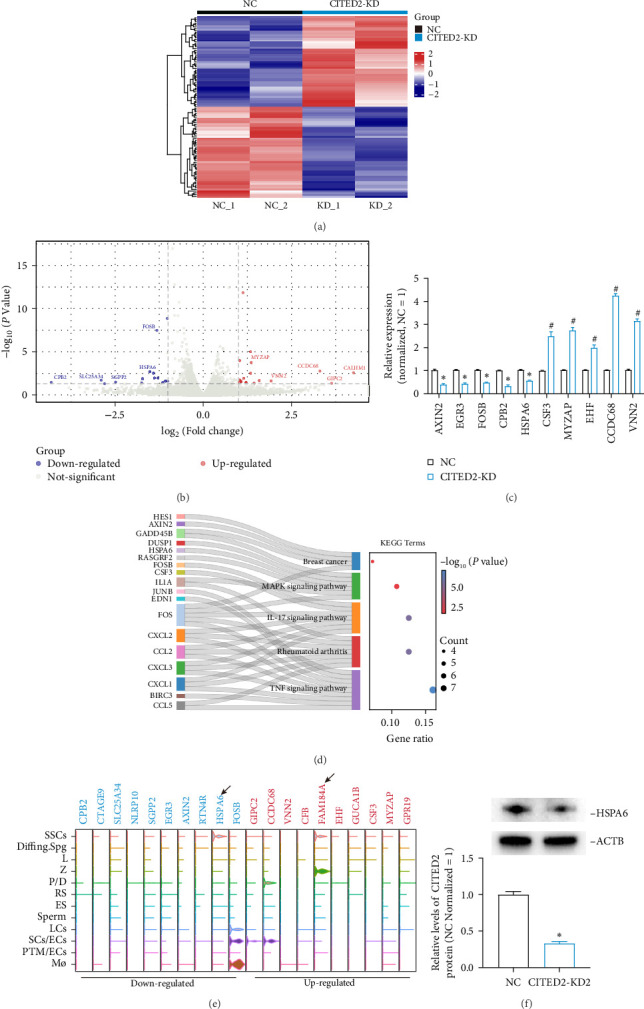
RNA sequencing screens for downstream targets of CITED2. (A) Heatmap showing the level of the Top 100 DEGs in each sample. The scaled gene expression levels are colored according to the Z score at right. (B) Volcano plot showing the distribution of all genes. Red represents significant upregulation, blue represents significant downregulation, and gray represents nonsignificant differences. DEGs of interest were labeled. (C) qPCR verified the expression levels of randomly selected DEGs. (D) The Sankey plot shows the Top KEGG terms and genes involved. (E) Violin plot demonstrating the levels of Top 20 DEGs in the testicular single-cell transcriptome. Arrows indicate genes that are highly expressed in SSCs. (F) Western blot detection of HSPA6 expression levels after CITED2 knockdown. *⁣*^*∗*^ represents *p* < 0.05, significantly downregulated compared to NC. # represents *p* < 0.05, significantly upregulated compared to NC.

To examine the downstream target genes of CITED2, RNA sequencing was utilized. After filtering out low-expression and duplicate genes, 15,882 genes were identified, with 24 genes significantly downregulated and 25 genes significantly upregulated. The top 100 DEGs were displayed using heatmaps (Figure 5A). Volcano plots illustrate the overall gene expression patterns, DEGs of interest were labeled (Figure 5B). Ten DEGs were randomly selected for validation using qPCR, and their expression levels aligned with the RNA sequencing results (Figure 5C). KEGG enrichment analysis was conducted on all DEGs, showing the top five KEGG terms and related genes in a Sankey plot. CITED2 downregulation impacted pathways, including breast cancer, MAPK, and IL-17 (Figure 5D). Additionally, the top 20 DEG expression was examined in the testicular monocytic transcriptome, revealing that HSPA6 and FAM184A were primarily localized in SSCs (Figure 5E). Based on western blot results, the HSPA6 protein level was downregulated following CITED2 knockdown (Figure 5F). Collectively, these results indicated that HSPA6 may be a potential target of CITED2.

### 3.6. HSPA6 Overexpression Diminished CITED2-Induced Phenotypic Changes

**Figure 6:**
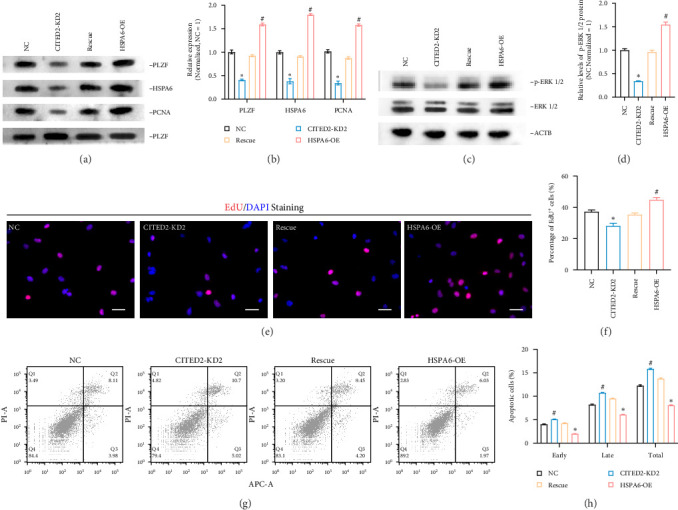
HSPA6 overexpression attenuates phenotypic defects caused by CITED2 downregulation. (A) Western blot was performed to detect the levels of PLZF, HSPA6, and PCNA. Four subgroups were included: NC group, CITED2 knockdown group (CITED2-KD2), Rescue group (CITED2-KD2+HSPA6-OE), HSPA6 overexpression group (CITED2-OE). (B) Bar graph showing the relative levels of each protein in A. (C) Western blot was performed to detect the expression level of phosphorylated ERK 1/2 (p-ERK 1/2) in the four groups. (D) Bar graph demonstrating the relative levels of p-ERK 1/2 in C. (E) EdU experiment detects DNA synthesis in cells in each group. Scale bar, 50 *μ*m. (F) Bar graphs show the proportion of EdU-positive cells in each group in E. (G) Flow cytometry was used to detect apoptosis in each group. (H) Bar graph showing the proportion of apoptotic cells in each group. *⁣*^*∗*^ represents *p* < 0.05, significantly downregulated compared to NC. # represents *p* < 0.05, significantly upregulated compared to NC.

To examine the influence of CITED2 on SSC proliferation and apoptosis through HSPA6, we conducted experiments involving CITED2 knockdown and HSPA6 overexpression to observe any potential corrective effects on cellular phenotypic defects. Four sample groups were established: NC (negative control), CITED2-KD (CITED2 knockdown), Rescue (concurrent knockdown of CITED2 and overexpression of HSPA6), and HSPA6-OE (HSPA6 overexpression). Western blot results showed that HSPA6 overexpression enhanced PLZF and PCNA expression and partially corrected protein downregulation resulting from CITED2 knockdown (Figure 6A,B). We also examined the expression of phosphorylated ERK1/2 protein (p-ERK1/2), a key molecule in the MAPK signaling pathway [34] and also reported to be essential for SSC proliferation and self-renewal [35]. The knockdown of CITED2 significantly reduced p-ERK1/2 levels, which aligned with RNA sequencing results. Overexpressing HSPA6 transiently activated p-ERK1/2 and counteracted the reduction induced by CITED2 knockdown, preventing p-ERK1/2 downregulation (Figure 6C,D). The EdU assay results demonstrated that HSPA6 overexpression mitigated the DNA synthesis defect caused by CITED2 knockdown (Figure 6E,F). Moreover, flow cytometry analysis of apoptosis showed that HSPA6 overexpression counteracted apoptosis triggered by decreased CITED2 levels (Figure 6G,H). These findings suggest that HSPA6 overexpression alleviates cellular phenotypic defects induced by CITED2 knockdown, indicating that HSPA6 is a downstream target of CITED2.

## 4. Discussion

SSCs are pivotal for spermatogenesis, providing sustained support for sperm production throughout a male's life [8]. In mice models, SSCs demonstrate the capacity to reinitiate spermatogenesis in recipient testes [36], presenting a potential solution for male infertility, particularly NOA. Nevertheless, our understanding of the regulatory mechanisms governing hSSCs is inadequate, impeding the advancement of stem cell-dependent therapeutic intervention for male infertility. Both intrinsic factors and the testicular microenvironment influence SSC behavior [37]. This microenvironment is instrumental in sustaining and directing SSC differentiation. Typically, SSC self-renewal and differentiation are modulated by Sertoli, Leydig, and peritubular myoid cells through their secretion of growth factors and the provision of structural frameworks [37]. Nevertheless, the precise molecular processes through which these cells orchestrate SSC behavior remain largely unknown.

CITED2 is a transcriptional cofactor that lacks DNA-binding motifs and regulates gene transcription by directly or indirectly interacting with transcription factors or cofactors [38]. It is essential for mouse embryo development, with deletion leading to embryonic lethality around day 10.5 [39]. CITED2 has been reported to be crucial for the development of organs such as the liver [40], lungs [41], heart [39, 42], and neural tube [43]. Additionally, it plays diverse roles in tumors and stem cells, being highly expressed in breast, lung, colon, and gastric cancers [44]. High expression of CITED2 promotes tumor growth and correlates with poor prognosis. In hematopoietic, neural, and embryonic stem cells (ESCs), as well as induced pluripotent stem cells, CITED2 is essential for self-renewal and survival [44, 45]. Knockdown of CITED2 severely impairs hematopoietic stem cell functionality (HSCs), and its lack triggers p53 activation, which in turn provokes HSC apoptosis, ultimately resulting in bone marrow failure [46]. CITED2 affects ESC self-renewal and survival by regulating Nanog, Klf4, and Tbx3 expressions through binding to EP300 [47]. Notably, overexpression of CITED2 maintains the ESC phenotype even when leukemia inhibitory factor is not present [48], which is a growth factor required for mouse SSC culture. These reports suggest that CITED2 is prevalent in stem and tumor cells and is necessary for cellular self-renewal, survival, and proliferation, which is generally consistent with our findings.

In our study, we comprehensively analyzed human testicular scRNA-seq data to simulate the developmental trajectory of SSCs. We identified several SSC-specific expressed genes, including ASB9, MAGBE2, SPOCD1, and TCF3, which have been manifested to be involved in SSC proliferation and apoptosis. However, the roles of other genes, including C19orf84, GNB2L1, and PFN1, in SSC fate determination remain unclear. Through experiments such as protein interaction database analysis and IP, we found that CITED2 and EP300 interacted, consistent with previous reports. Additionally, CITED2 is predicted to bind to FOXO1, a protein essential for SSC self-renewal and proliferation [14]. Further investigation is needed to determine if these proteins interact and regulate SSC fate. Due to poor antibody specificity, the EP300 antibody did not effectively enrich proteins when used in IPAs. More experiments are needed to verify the enrichment of CITED2 by the EP300 antibody.

Through RNA sequencing, we identified several genes regulated by CITED2. While CITED2 is known to regulate gene expression by binding to EP300, further evidence is required to confirm that the CITED2-EP300 complex directly regulates HSPA6. HSPA6, a member of the HSP70 family, is a partially conserved inducible protein in mammals [49]. It has been linked to the promotion of various tumor growths and is related to poor prognosis [49]. HSPA6 enhances gastric cancer cell proliferation by increasing cyclinB1 and YAP levels [50]. Although there are limited functional studies of HSPA6 in stem cells, our findings parallel those in tumor cells, where HSPA6 is crucial for cell proliferation and survival. By conducting an enrichment analysis of all DEGs, we focused on those enriched in the MAPK signaling pathway and further validated the role of HSPA6. However, genes in other pathways may also influence the regulation of SSC function by CITED2, necessitating additional studies for confirmation. Additionally, rescue experiments demonstrated that an increase in HSPA6 partially restores the phenotypic defects induced by CITED2 deficiency. Specifically, the concurrent downregulation of HSPA6 and CITED2 did not significantly exacerbate the attenuation of cell proliferation compared to CITED2 knockdown alone (data not shown). Whether this effect is due to other compensatory mechanisms that compensate for the function of HSPA6 requires further investigation.

While we have confirmed the role of CITED2 in hSSCs *in vitro*, further research is required to elucidate its function within the testis in vivo. Additionally, exome sequencing conducted on azoospermic patients did not reveal any significant CITED2 mutations. This may be attributed to the crucial role of CITED2 in embryonic development, where its absence is known to be lethal. Future studies will focus on understanding the impact of CITED2 on spermatogenesis by developing a CITED2 testicular conditional knockout mouse model.

## 5. Conclusions

Our research identified a predominant CITED2 expression in hSSCs through scRNA sequencing analysis alongside IHC. In these cells, the knockdown of CITED2 led to a decrease in HSPA6 expression and inhibits MAPK signaling, which subsequently affects cellular self-renewal and survival (Supporting Information Figure S1). These data provide novel perspectives into SSC developmental regulation, thereby offering a theoretical foundation for treating male infertility.

## Data Availability

The data that support the findings of this study are available from the corresponding author upon reasonable request.

## Supporting Information

Supporting Information Supporting Information Figure S1: Graphic abstract of this study. Table S1: The primers used for qPCR. Table S2: Antibodies applied in Western blots, immunofluorescence.
