# Supplementary material for: CITED2 Binding to EP300 Regulates Human Spermatogonial Stem Cell Proliferation and Survival Through HSPA6
Source: Stem Cells Int. 2025 Apr 24;2025:2362489. doi: 10.1155/sci/2362489 (PMC12045681; doi:10.1155/sci/2362489)
Supplement: Supporting Information — Figure S1: Graphic abstract of this study. Table S1: The primers used for qPCR. Table S2: Antibodies applied in Western blots, immunofluorescence. [file 2362489.f1.pdf]

Supplementary information

CITED2 Binding to EP300 Regulates Human SSC Proliferation and Survival through MAPK Pathway and HSPA6

Supplemental Data:
1Figure
2Tables

Supplemental Figure

Figure S1. Graphic abstract of this study

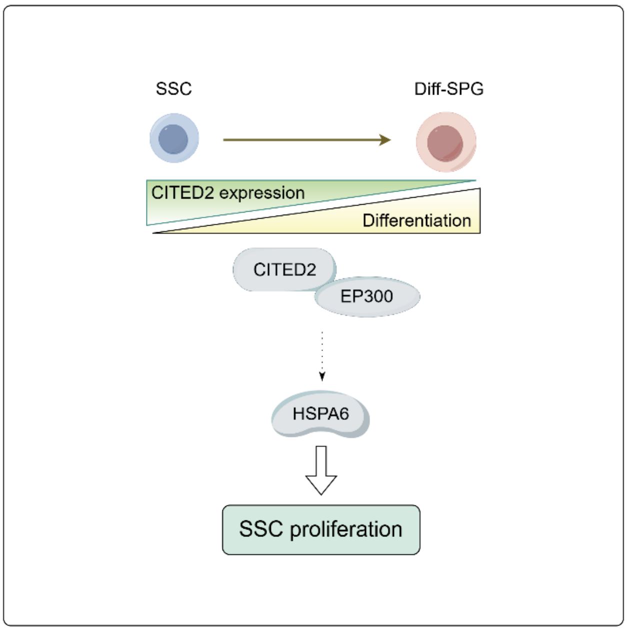

Supplemental Tables

Table S1. The primers used for qPCR

Genes	Sequence
CITED2	**F:** CCTAATGGGCGAGCACATACA
	**R:** GGGGTAGGGGTGATGGTTGA
AXIN2	**F:** TACACTCCTTATTGGGCGATCA
	**R:** TTGGCTACTCGTAAAGTTTTGGT
EGR3	**F:** GACATCGGTCTGACCAACGAG
	**R:** GGCGAACTTTCCCAAGTAGGT
FOSB	**F:** GCTGCAAGATCCCCTACGAAG
	**R:** ACGAAGAAGTGTACGAAGGGTT
CPB2	**F:** GAACTGTCTCTAGTAGCCAGTGA
	**R:** TGCCCAAATCATAGATCCAATCG
HSPA6	**F:** GATGTGTCGGTTCTCTCCATTG
	**R:** CTTCCATGAAGTGGTTCACGA
CSF3	**F:** CTTGTGGCCTATAACTCAGCC
	**R:** CCCACTCAATCACATAGCCCT
MYZAP	**F:** TGGAACCTACTGAACGACCTC
	**R:** ATCCAAACGATCCATGTGGCA
EHF	**F:** CAGTGCAGTAGTGACCTGTTC

	**R:** CTGTGCTACCATAGTTGGTGTC
CCDC68	**F:** TTGTGATTCACTCCCAACATCAG
	**R:** GACAGCCTTAGATACCTTTGTCC
VNN2	**F:** CAGGGTGCTCGAATCATTGTG
	**R:** CACGGAATCCAGTTCACCTGA

Table S2. Antibodies applied in Western blots, immunofluorescence

Antibodies	Source	Dilution	Incubation
Western blot			
CITED2	Abcam cat# ab314758	1:1000	12h at 4°C
PLZF	SantaCruz cat#sc-28319	1:1000	12h at 4°C
PCNA	Abcam cat#ab29	1:500	12h at 4°C
ERK1/2	Promab cat#30014	1:1000	12h at 4°C
p-ERK1/2	Zenbio cat#R380698	1:1000	12h at 4°C
THY1	Abcam cat#ab92574	1:1000	12h at 4°C
CASP3	Abcam cat#ab32042	1:1000	12h at 4°C
ACTB	Promab cat#20270	1:2000	12h at 4°C
EP300	LSbio cat# LS-B6081-50	1:1000	12h at 4°C
HSPA6	Abcam cat#ab ab212044	1:1000	12h at 4°C
Immunofluorescence			
CITED2	Abcam cat# ab314758	1:50	16h at 4°C
GFRα1	R&D cat#AF560	1:25	16h at 4°C
KIT	R&D cat#AF332	1:25	16h at 4°C
PCNA	Abcam cat#ab29	1:50	16h at 4°C
EP300	LSbio cat# LS-B6081-50	1:50	16h at 4°C
